# How online sexual health services could work; generating theory to support development

**DOI:** 10.1186/s12913-015-1200-x

**Published:** 2015-12-05

**Authors:** Paula Baraitser, Jonathan Syred, Vicki Spencer-Hughes, Chris Howroyd, Caroline Free, Gillian Holdsworth

**Affiliations:** Kings Centre for Global Health, Kings College London, London, UK; Department of Sexual Health and HIV, Kings College Hospital NHS Foundation Trust, London, UK; Lambeth and Southwark Public Health Directorate, London, UK; SH:24, London, UK; Department of Public Health, London School of Hygiene & Tropical Medicine, London, UK

**Keywords:** Sexually transmitted diseases, Testing, Internet, Programme theory, Theory of change

## Abstract

**Background:**

Online sexual health services are an emerging area of service delivery. Theory of change critically analyses programmes by specifying planned inputs and articulating the causal pathways that link these to anticipated outcomes. It acknowledges the changing and contested nature of these relationships.

**Methods:**

We developed two versions of a theory of change for an online sexual health service. The first articulated the theory presented in the original programme proposal and the second documented its development in the early stages of implementation through interviews with key programme stakeholders.

**Results:**

The programme proposal described an autonomous and empowered user completing a sexual health check using a more convenient, accessible and discreet online service and a shift from clinic based to online care. The stakeholder interviews confirmed this and described new and more complex patterns of service use as the online service creates opportunities for providers to contact users outside of the traditional clinic visit and users move between online and clinic based care. They described new types of user/provider relationships which we categorised as: those influenced by an online retail culture; those influenced by health promotion outreach and surveillance and those acknowledging the need for supported access.

**Conclusions:**

This analysis of stakeholder views on the likely the impacts of online sexual health services suggests three areas for further thinking and research.

1. Co-development of clinic and online services to support complex patterns of service use.
2. Developing access to online services for those who could use them with support.
3. Understanding user experience of sexual health services as increasing user autonomy and choice in some situations; creating exclusion and a need for support in others and intrusiveness and a lack of control in still others.

This work has influenced the evaluation of this programme which will focus on; mapping patterns of use to understand how users move between the online and clinic based services; barriers to use of online services among some populations and how to overcome these; understanding user perceptions of autonomy in relation to online services.

## Background

Increasing demand for sexual health services, limited resources for sexual health care [1] and new tests that use non-invasive samples have driven investment in online sexual health services [2]. These services enable users to register on a secure website and order sexual health tests to be sent to their home. Self-taken samples are posted by the user direct to the laboratory with results delivered by text message to mobile phones. Treatment can be delivered by post after a telephone consultation to exclude contraindications to the relevant medication [3]. Additional, personalised, sexual health information and health promotion can be provided through the website, text messages or online chat.

Online sexual health services are thought to be less expensive than similar services delivered in clinic settings [4] and to increase access, at least for some groups [2, 5]. There is evidence to suggest a demand for these services with 50,000 online tests for genital chlamydia infection delivered annually within the English National Chlamydia Screening Programme [2]; new services for home HIV testing [6] and a growing online private market in the diagnosis and treatment of sexually transmitted infections [7].

In 2013 we started developing and evaluating an online sexual health service linked to a telephone and clinic-based service in an area of South East London with a young, multi-ethnic population experiencing high levels of socio-economic deprivation and with very high levels of sexual ill health. This programme proposes a web based sexual health service with a linked telephone service and direct referral into local clinics when required. The service is planned to evolve through small-scale prototype development and testing. As recommended for the evaluation of complex interventions we sought to generate a conceptual model for the intervention, link this to the published evidence and the local context and then monitor its development during programme implementation [8].

We developed a theory of change to articulate the conceptual model underpinning the programme and its development. Theory of change critically analyses new programmes by articulating the causal pathways that link specified inputs to anticipated outcomes. It acknowledges the need to test these relationships for new programmes and their development during implementation [9–12]. Theory of change is also a reflective process and dialogue among stakeholders that makes explicit underlying assumptions of how and why change might happen as a way of supporting programme innovation and adaptation [9–12]. We followed Vogel [11] and Van Belle [12] in using a simplified theory of change methodology to ensure theory generation fast enough to influence service development. We analysed the programme funding application and stakeholder views early in the development process to generate two initial theories of change that could be considered part of a single evolving theory of change. We focused on the first three stages of the Theory of Change process described by the Centre for the Theory of Change [13]. These are:

1. Identifying long term goals
2. Backwards mapping and connecting outcomes
3. Identifying assumptions

This process informed our evaluation strategy for the programme by identifying key outcomes, process indicators and assumptions that required testing.

## Methods

Van Belle et al. [12] recommend three data sources for theory of change construction: a) the initial programme funding application; b) the views of programme stakeholders including potential programme recipients and c) evidence from similar or related interventions.

The proposed long term goals of the programme were identified from the original funding bid, developed by the two London borough public health departments and two NHS foundation trusts providing specialist sexual health services. We (PB and JS) read and re-read this document to identify the planned inputs and anticipated outputs described in the document and the steps that connected them. We developed a series of diagrams to describe these linkages and the assumptions that underpinned them. We imported the document into qualitative data analysis software Nvivo (NVivo; QSR International Pty Ltd. Version 10, 2012) and coded this material using our coding frameworks based on early versions of our theory of change diagrams to check the relationship between our initial theory of change and the text of the bid. We completed several cycles of causal model development and modification to ensure that our diagram accurately reflected the information in the bid. Finally we identified the key assumptions that underpinned the linkages in our causal model as important areas for evaluation.

Having developed an initial version of the theory of change from the programme funding bid we then repeated a modified version of this process during programme implementation using interviews with stakeholders as our data source.

We recruited our interview participants to reflect the full range of stakeholders for the project including;

- Four clinicians in local services (doctors, nurses, client support workers)
- Three senior managers within clinical services
- Three senior managers within local government, public health and commissioning
- Four potential service users sampled to include individuals from the populations most at risk of sexual ill health in the local area; men who have sex with men, young people, and those from black and minority ethnic groups

All of the 14 stakeholders who were invited to complete individual interviews accepted our invitation. Each participant provided written informed consent. Interviews began with the interviewer describing the planned service inputs (an online sexual health service with a linked telephone service and direct referral into local clinics when required). These were listed on the left hand side of a large sheet of paper. Participants were then asked to list all of the outcomes, both positive and negative that they anticipated from this input. These were listed on the right hand side of the paper. By asking what needed to happen for the input to lead to the anticipated outcome and why this needed to happen participants were encouraged to map this relationship and to reflect on the assumptions that underpinned it [13]. As this process continued the necessary stages for movement from the input to the outcome were added to the diagram. Interviews continued until all the outcomes suggested had been discussed. All interviews were recorded and fully transcribed.

Our qualitative analysis used the framework approach [14], a matrix based analytic method that organises data according to key themes, sub themes and emergent categories [15]. Two researchers (JS and PB) familiarised themselves with the data (the original funding application and the stakeholder interviews) through reading and re-reading the texts. Through discussion within the evaluation team (PB, JS, VSH) we developed coding categories that reflected emerging themes and refined these through three rounds of coding and modification (see Table 1 for final coding categories). Data was coded separately by four different researchers using the qualitative data analysis software Nvivo (NVivo; QSR International Pty Ltd. Version 10, 2012). Coding consistency was checked through review of all coding by JS. Differences in coding were resolved through discussion. A second theory of change was developed on the basis of this analysis.

**Table 1:** Final coding categories

Coding category	Sub-codes	Link to theory of change diagram
Changing user-provider relationships	• User empowerment/disempowerment • New opportunities for technology mediated support • Creating expectations of an online retail service within public sector • Loss of face to face contact	• Changes in user options and responsibilities • Expectations of provider driven, technology mediated contact • Targeting the online service beyond the autonomous consumer
Impact on clinics	• Change to clinic systems • Changes to interaction between sexual health services • Changes to clinic staffing	• New patterns of service use • Changes in clinic processes, staff training • Potential for linking development of clinic based and online services
Impact on STIs at population level	• Increased STI testing • Changes in sexual behaviour • Changes in access to sexual health promotion • Changes in access to services between different population groups	• Reduced STI transmission in the population
Changes in cost of service provision across the whole sexual health economy	• Changes in cost per test • Changes in demand triggered by online service including creation of inappropriate demand • Movement between services/dual use • Changes to clinic capacity	• More cost effective sexual health services

Ethical approval for this research was granted by King’s College London research ethics committee (Ref: BDM/13/14-42).

## Results

### The initial theory of change

**Fig. 1:**
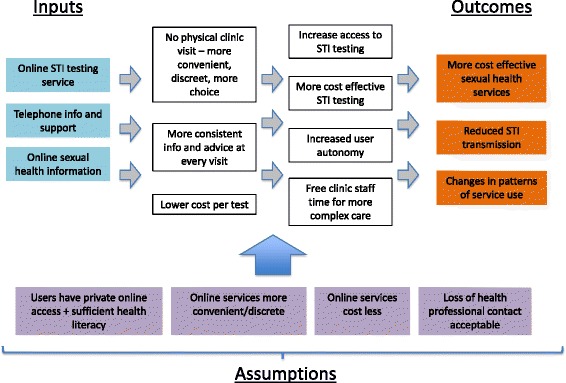
Theory of change for an online testing service for sexually transmitted infections as articulated in the funding proposal

The theory of change developed from the programme funding application articulated a causal chain with more convenient and discreet sexual health testing leading to increased uptake of testing and treatment, coupled with consistent health promotion advice to generate the final outcome of reduced STI transmission (Fig. 1). It referenced user autonomy in making decisions about their care and in navigating their preferred route through services. It predicted changes in patterns of service use with some users moving from clinic based to online services. It anticipated a direct translation of the pattern of service use common in clinic to the online service, that is a single service visit to complete a sexual health check and it predicted that online services would release capacity in clinics for those requiring complex care. These changes were predicted to increase capacity without an increase in cost. This theory of change is built on a number of assumptions about online services;

- that potential users have private internet access and sufficient health literacy to use an online facility
- that the online service is more convenient and discrete than use of clinical services, at least for some people; that the pattern of service use replicates
- that online services cost less than clinic based services
- that the loss of health professional contact associated with online services is acceptable to users.

### The second theory of change developed from stakeholder interviews

The theory of change developed from the stakeholder interviews supported the idea of online services as more convenient and more discrete (see Table 2 below) but challenged and developed the assumptions in the programme bid.

**Table 2:** Stakeholder views on key assumptions within the initial bid

Assumption	Elements/dimensions indentified	Examples from data
Increased convenience	Long waiting times and risk of being turned away if walk in service is full	*..when reception sit there and say, “We’ve got no more tickets,” or, “The waiting time is currently three hours, roughly,” I think we could then say to someone, “Look, if you’re not worried about anything, that (the online service) is your other option”. (Clinic staff)*
	Inconvenient opening times or location.	*There will be people who will never darken the doors of a GU clinic because they’re …….not open enough hours to meet their working schedule (Clinic staff)*
Increased discretion	People who fear being seen using a clinic by someone.	*If you can do it all remotely and without anybody knowing or seeing you waiting outside a sexual health clinic and going, “Oh, what are you doing here?” then I think it’s going to be absolutely brilliant (User)*
	Embarrassment discussing issues with clinician	*…the whole process of making appointments and going and seeing, feeling embarrassed, having to come and see somebody old and crusty like me. (Clinical, primary care)*

The participants predicted new patterns of service use and new forms of user/provider interaction. Rather than a straightforward shift of activity from one service to another they thought users would choose to move between services for example complementing a clinic visit with online information or an online sexual health check with a visit to the clinic for reassurance or support.

*I think, it’s also something you can do at your own pace. So, say, if you had five minutes, you could quickly go online and have a look and you don’t have to decide immediately. I think there’s a certain pressure that comes with going to a clinic or calling up as well. (User)*

This more complex view of how users might choose to use services questioned the simple cost effectiveness analysis proposed in the initial bid. Stakeholders described the additional responsibilities associated with self management, loss of support/reassurance/empathy that might be part of a clinical consultation and missed opportunities to identify additional unmet need, for example, advice and referral for those experiencing intimate partner violence.

*‘I’ve tried to find out information online, for clients here, and there is some information on NHS Direct, but unless you can actually spell chlamydia or gonorrhoea, or syphilis; it’s actually quite hard to find information”.*

They described new roles for clinic staff with provider driven, technology mediated, contacts such as reminders to take treatment or notify partners or follow up offers of tests or support. They emphasised the potential inequity of access to online services and the possibilities for providing additional remote support for those who might be able to use the service with help. These new approaches to service use were associated with descriptions of user/provider relationship. We summarised these as an ‘online retail approach’ that emphasises patient autonomy and choice; a ‘public health promotion/surveillance’ approach that emphasises outreach to support behaviour change or monitoring of service access and a ‘supported care’ approach that references a need for health care professional mediated support during use of the online service (Table 3).

**Fig. 2:**
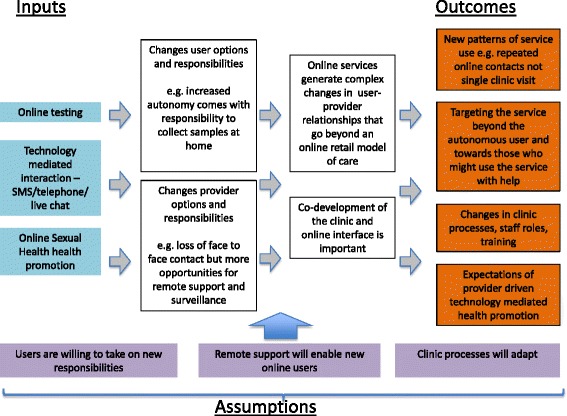
The theory of change for an online sexual health service developed from the stakeholder interviews

Based on the stakeholder interviews we developed a second theory of change that incorporates this thinking (see Fig. 2). This emphasises the new opportunities and new responsibilities for both users and service providers that come from the introduction of online sexual health services. It references the movement between online and clinic based care, the new responsibilities that go with self care, an increase in remote, service driven contacts and alternative ways of conceptualising the online service user. The modified theory of change has three further assumptions essential to these new causal pathways; (i) that users are willing to take on new responsibility for their own sexual health care; (ii) that remote support will enable new online users; (iii) that clinic processes will adapt to the new online service provision.

**Table 3:** New approaches to user/provider relationships for STI testing services

Public health promotion/surveillance	*“Gosh, I can manage my own care this way, and I can do other things too.” It is sort of educational. It may well enhance self-esteem by doing that and remaining healthy, or living a healthier lifestyle, and have knock-on benefits elsewhere. You’d hope”.* (Executive) *for all positives we say to them, “This is when you should re-test,” and we can then send people packages or invite them to pick up packages for retesting at that time (clinic staff)*
Supported health care approach	*“rather than just treat them and kick them out of the website, I think there has to be some thought given to information, education, being able to answer people’s questions quickly, some empathy on a telephone line for the asymptomatic patients I see …..they don’t understand what a symptom is. You ask them if they have unusual discharge, pain, the usual stuff, and because that’s layman terms to them they’re like, “Oh yeah, I have that”. (Clinic staff)* *‘You could develop a … pathway that gave human contact maybe at the beginning and at the end and telephone contact two or three times through the pathway depending on need …let me show you how to access it. Can you find that page? OK, click in the bottom left-hand corner’ (Clinical Manager).*
Online consumer approach	*“The patient is the customer and is absolutely at the heart of that brand and I think it changes the power a little bit. It’s the power of patients to be able to control the journey that they have with their own healthcare”. (executive)*

## Discussion

The development of these two versions of the theory of change for this programme contributes to service development, implementation and evaluation. It identifies the development of support packages to enable online service use as a priority for service development; the co-development of online and clinic based services as a priority within the implementation strategy and the need to document new patterns of service use as clients move between clinic and online options as a priority for evaluation.

The rationale for the development of online services as described in the two versions of our theory of change is based on a number of key assumptions. The architects of the original bid assume; private internet access, health literacy, an online service that is convenient and discrete, online services that are cost effective and the acceptability of loss of a consultation. These are supported by evidence from both similar interventions and other relevant sources. National statistics show that 76 % of UK adults accessed the internet daily in the 2014 and 96 % of 16 to 24 year olds access the internet via mobile device [16]. There is no data on health literacy levels in England [17] but a 2011 national skills survey of England found 15 % of the adult population (5.1 million) have very low levels of literacy (Entry level 3 or below) [18]. There is evidence that online sexual health services are popular in the UK [7] with 59,000 online tests taken within the English National Chlamydia Screening Programme in 2010 [2]. Randomised trials have found higher or equal uptake rates for home based testing when compared with clinic-based provision [19] and cross-sectional and qualitative studies have demonstrated convenience [5, 20–24]. It is less clear that online testing is considered discrete [5] and we found no data on the acceptability of loss of health professional contact although qualitative studies reported this as a disadvantage particularly with testing for HIV [20, 21]. Chlamydia and Gonorrhoea screening programs using home testing have been shown to be cost-effective when compared to clinic based screening [25–27] although this is sensitive to uptake and return rates [23, 27–29] particularly for HIV tests [30].

The modified theory of change has three further assumptions essential to these new causal pathways; that users are willing to take on new responsibility for their own sexual health care; that remote support will enable new online users and that clinic processes will adapt to the new online service provision. The stakeholder interviews echo issues raised in the published literature on the potential of internet services to disproportionately enable some users to take on increased responsibility for their own health care [31, 32]. Those without these resources have potentially reduced access to the convenience of online care. The published literature documents a range of responses to this potential inequity including free internet access at strategic sites, improved readability and cultural acceptability of online health information, skill development for online access or additional support from a care manager [32–36]. There is evidence from collaborative care models in mental health care that patient facing online services are less effective when they are provided as stand alone interventions rather than in the context of a relationship with a counsellor or healthcare provider [37]. Human support is often important for patient engagement and this is particularly important where clients have some health literacy issues [35].

The stakeholder interviews document what is potentially lost through use of online services and while suggesting that everyone should have access also suggests that not everyone will want to have access. This echoes the critiques of telemedicine and e-health that describe the potential loss of agency that comes from allowing digital health technologies into the home [38]. The revised theory of change incorporates ideas of how healthcare technologies help to construct particular types of users and provider/user relationships. The online service introduces the idea of the autonomous patient with more options to distance him/her from the clinic space controlled by the health care provider. However it simultaneously reduces the distance between health care provider and user enabling health care provider access to the users’ private space as email and SMS reminders to access care or change behaviour become part of the online package [38]. Design of online services to maximise utility for a wide range of users suggests strategies including computer mediated human support will enable new online users [31, 33–37, 39].

Finally the stakeholder interviews suggest that online service introduction should be considered as part of a dynamic sexual health economy and not a stand-alone service. The clinic is likely to experience changes in its users, the problems they present and the clinic staff and processes available to respond as clients move between the online, telephone and face to face service. This is not a bolt on service but a change to the whole system of sexual health service provision and close working between the different elements of the system will be essential. A 2014 review of the literature suggests that co-development of online and clinic based services has received little attention to date but is an emerging area of focus in the development of online health services [39, 40].

## Conclusion

By documenting the development of the theory of change during implementation we identified three elements of online services that would benefit from further research.

1. Co-development of clinic and online services to support complex patterns of service use.
2. Developing access to online services for those who could use them with support.
3. Understanding user experience of sexual health services as increasing user autonomy and choice in some situations; creating exclusion and a need for support in others and intrusiveness and a lack of control in still others.

This work has influenced the evaluation of this programme which will focus on; mapping patterns of use to understand how users move between the online and clinic based services; barriers to use of online services among some populations and how to overcome these; understanding user perceptions of autonomy in relation to online services.
